# Accuracy of digital impressions for implant fixed dental prostheses in partial and complete maxillary edentulous arches: effect of intraoral scanners and implant position

**DOI:** 10.1186/s12903-026-07887-6

**Published:** 2026-03-11

**Authors:** Marwa Emam, Mostafa Aldesoki, Christoph Bourauel

**Affiliations:** 1 Department of Fixed Prosthodontics, Faculty of Dentistry, Ain Shams University, Cairo, Egypt; 2 Department of Fixed Prosthodontics, Faculty of Dentistry, Galala University, Suez, Egypt; 3 Oral Technology, Dental School, University Hospital Bonn, Bonn, Germany

**Keywords:** Digital impression, Intraoral scanners (IOSs), Trueness and precision, Implant fixed denture prostheses, All-on-4

## Abstract

**Background:**

There is a lack of comprehensive comparative data assessing the accuracy of multiple intraoral scanners (IOSs) across both partially and completely edentulous cases, particularly in the presence of variable implant positions. The aim of this study was to assess the effect of IOSs and implant positions in 4-unit and All-on-4 restorations on the trueness and precision of digital impressions.

**Methods:**

Two maxillary resin models were fabricated: a partially edentulous arch planned for a 4-unit fixed dental prosthesis (FDP) with implants at the canine and first molar sites, and a completely edentulous arch for an All-on-4 FDP. Each model was scanned (*n* = 10 each) using five IOSs: Omnicam AF, CS 3800, Trios 3 A, Medit i500, and Primescan AC. Scan data were analyzed using Geomagic Control X to assess trueness and precision. Statistical analysis was performed using three-way ANOVA, followed by two-way ANOVA and post hoc testing (*p* < 0.05).

**Results:**

Regarding trueness in the 4-unit FDP group, CS3800 exhibited the highest RMS in anterior and posterior implants. In the All-on-4 group, Trios and Medit i500 showed the lowest RMS in anterior implants, and Medit i500 showed the lowest RMS in posterior implants. CS3800 showed the highest RMS in both the implant positions. Regarding precision in the 4-unit FDP group, Trios and Primescan achieved the lowest RMS. CS3800 and Medit i500 showed the highest RMS in posterior implants. In All-on-4, Omnicam showed the least RMS in anterior implants, while Primescan the least in posterior implants. All scanners showed less precision in posterior implants in both models.

**Conclusions:**

Under controlled in vitro conditions, differences in trueness and precision were observed among intraoral scanners, depending on implant position and restoration type. In All-on-4 restorations, Medit i500 showed the highest trueness. Whereas, in 4-unit FDPs, Trios 3 and Primescan exhibited the highest precision. Anterior and posterior implant positions exhibited varying levels of trueness and precision deviation across scanners.

**Clinical significance:**

Intraoral scanner accuracy is influenced by implant position and prosthesis configuration, with posterior implant positions generally exhibiting reduced precision under in vitro conditions. These findings represent statistically significant differences observed within an experimental model and do not establish scanner ranking, clinical acceptability thresholds, or in vivo scanner suitability.

**Graphical Abstract:**

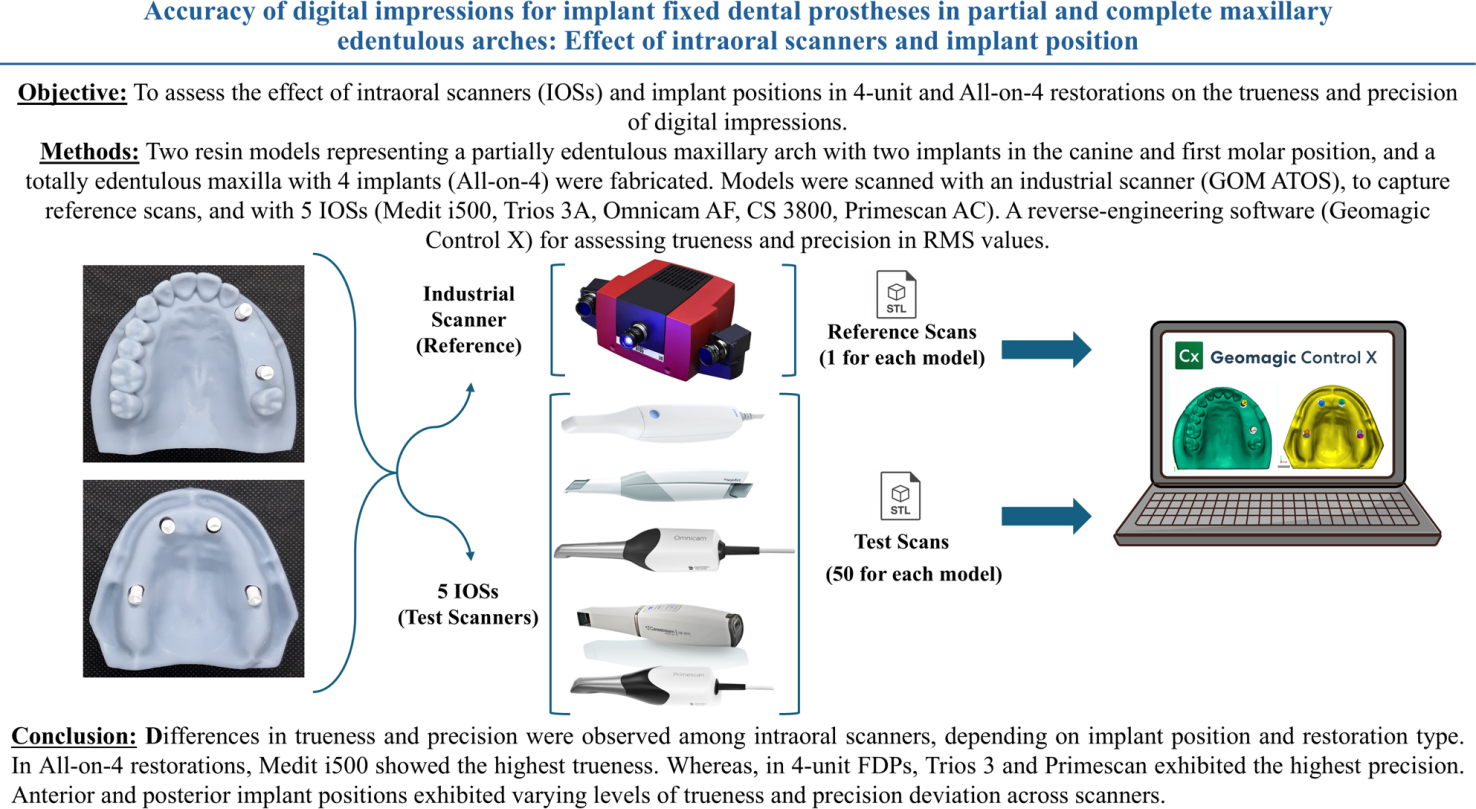

**Supplementary Information:**

The online version contains supplementary material available at 10.1186/s12903-026-07887-6.

## Background

There has been a paradigm shift in the workflow for dental prosthetics in the past few years. Intraoral scanners (IOSs) have been used extensively to digitize dental arches into 3-dimensional (3D) virtual models for the computer-aided design and manufacturing (CAD/CAM) of various dental restorations and prostheses, in place of dental impressions and gypsum models [1, 2]. This approach offers real-time visualization, improved efficiency, and simplified data transfer to the dental laboratory [3]. Intraoral scanners capture dental surfaces using optical imaging technologies to generate 3D digital models [4, 5]. Current IOSs employ different optical imaging principles, such as triangulation-based systems and confocal or parallel confocal technologies. These approaches differ in how surface data are captured and reconstructed, which may influence accuracy, particularly in extended scanning spans and implant-supported restorations [6, 7].

To obtain high-quality digital impressions, an IOS must demonstrate both trueness and precision [8]. Trueness refers to how closely a scan reflects the actual dimensions of the object being measured, while precision describes the scanner’s ability to consistently reproduce the same results across multiple attempts. Ideally, both trueness and precision are essential components of IOS accuracy [3, 8–10].

Paulo Malo developed the All-on-4 concept in 1998 to deliver an immediate loading temporary prosthesis and restore a fully edentulous arch with just four implants [11, 12]. Two implants are positioned axially in the anterior region of the arch as part of the All-on-4 concept, and two posterior implants-one on each side-are positioned anterior to the maxillary sinus and tilted distally to improve prosthesis support [13]. Typically, the two posterior implants are often positioned at a 30- or 45-degree angle at the second premolar/molar region [14]. By using slanted implants, cantilever forces in the posterior region are reduced, and a 12-unit prosthesis can be used regardless of the anterior implant location.

Accurately transferring the 3D implant location from the patient’s mouth to the master model or prosthesis design software is the most crucial stage in creating an implant-supported, long-lasting prosthesis. An incorrect transfer of the implant position causes the prosthesis to compromise the passive fit of the prosthesis and can lead to biomechanical concerns, including screw loosening and bone resorption [15–18]. Owing to the limited IOS field of view and distortions in the frame-stitching process imposed by the absence of anatomical landmarks and increased scanning distance, a complete arch implant scan for an All-on-4 configuration is still quite challenging [19, 20]. A restricted number of reference points might end up in duplicate data, misaligned scan segments, or inaccurate software interpretation. Moreover, when recapturing missing sections, reflective edentulous ridges can cause additional errors [19, 21].

Despite the growing body of research on IOS performance, relatively few studies have investigated the accuracy of digital impressions of implant-supported restorations using intraoral scanning for acquisition [7, 8, 12, 14, 17–19]. The available evidence predominantly concerns single-implant restorations, for which favorable outcomes have generally been reported [18]. In contrast, substantially fewer investigations have addressed multi-implant and extended restorations, where existing data suggest that capturing accurate digital impressions may be more challenging, particularly for full-arch restorations [14, 18].

Consequently, limited data are available comparing the trueness and precision of multiple IOSs across both partial and complete edentulous situations, with varying implant positions [7]. This gap in literature constrains the evidence-based selection of IOSs for different clinical situations, underscoring the need for further investigation. Therefore, it was the aim of this in vitro study to evaluate and compare the trueness and precision of five IOSs in capturing digital impressions for implant-supported FDPs in partially and completely edentulous arches, considering the influence of implant position. The null hypothesis was that no significant differences in trueness or precision would be detected among the tested IOSs, irrespective of implant position, in either partially or completely edentulous arches.

## Methods

### Ethical clearance and study design

Under exemption number (FDASU-RecER062517), the Research Ethics Committee of the Faculty of Dentistry, Ain Shams University (FDASU) approved this study. Two maxillary resin models were fabricated: a partially edentulous arch planned for a 4-unit fixed dental prosthesis (FDP) with implants at the canine and first molar sites, and a completely edentulous arch for an All-on-4 FDP. Each model was scanned using industrial reference scanner and five test IOSs (*n* = 10 each): Omnicam AF, CS 3800, Trios 3 A, Medit i500, and Primescan AC. Digital impression were analyzed using reverse engineering software to assess trueness and precision before statistical analysis was conducted.

### Models fabrication

Two individual models, each reflecting a different clinical situation in the maxillary arch, were created. For the first situation, a typodont (Nissin Dental) was modified to represent a partially edentulous maxilla with missing maxillary right canine, premolars, and first molar, which was planned to be restored with Fixed Denture Prosthesis (FDP) on two implants in the canine and first molar positions. The previously specified missing teeth were removed from the typodont model, and their sockets were blocked with wax to simulate an edentulous ridge. For the second clinical case, an anonymous gypsum model of a completely edentulous maxilla was used and it was planned to be restored with an All-on-4 FDP, with the implants to be placed in lateral incisors (parallel) and first molars (45^○^) positions.

Subsequently, the models were scanned and the stereolithography (STL) files were imported into 3-matic software (version 17.0; Materialise) for planning the implant placement. STL files of implant bodies 4.2 × 13 mm (tioLogic ST, Dentaurum Implants) were virtually inserted in the planned positions using the “Boolean subtraction” function to create the space for the implant body in the model after 3D printing. Finally, the models were printed using dental model resin (Savoy dental model resin; Savoy digital systems, Printfy 3D) after exporting STL files to a 3D printer (Anycubic photon S). Following printing, dental implants were inserted and glued in the specified positions and titanium base abutments (CAD/CAM Ti base M, Dentaurum) size M were screwed (30 Ncm) to the implants before the scan bodies for TiBase (Dentsply Sirona) were attached.

### Sample size

A power analysis was conducted based on the results of a previous study [18]. By adopting (0.05) alpha (α) level, (0.05) beta (β), (power = 95%), and (2.648) effect size (f); the predicted minimum sample size (n) was 5 scans per scanner. To enhance statistical power, sample size was doubled for a greater effect to (*n* = 10) scans per scanner per model. The power analysis was based on main effects reported in prior studies. While the sample size was sufficient to detect large effects, interaction effects in multifactorial designs may require larger sample size and should be interpreted cautiously. G*Power version 3.1.9.7 [22] was used for sample size calculation.

### Digital impressions

**Fig. 1:**
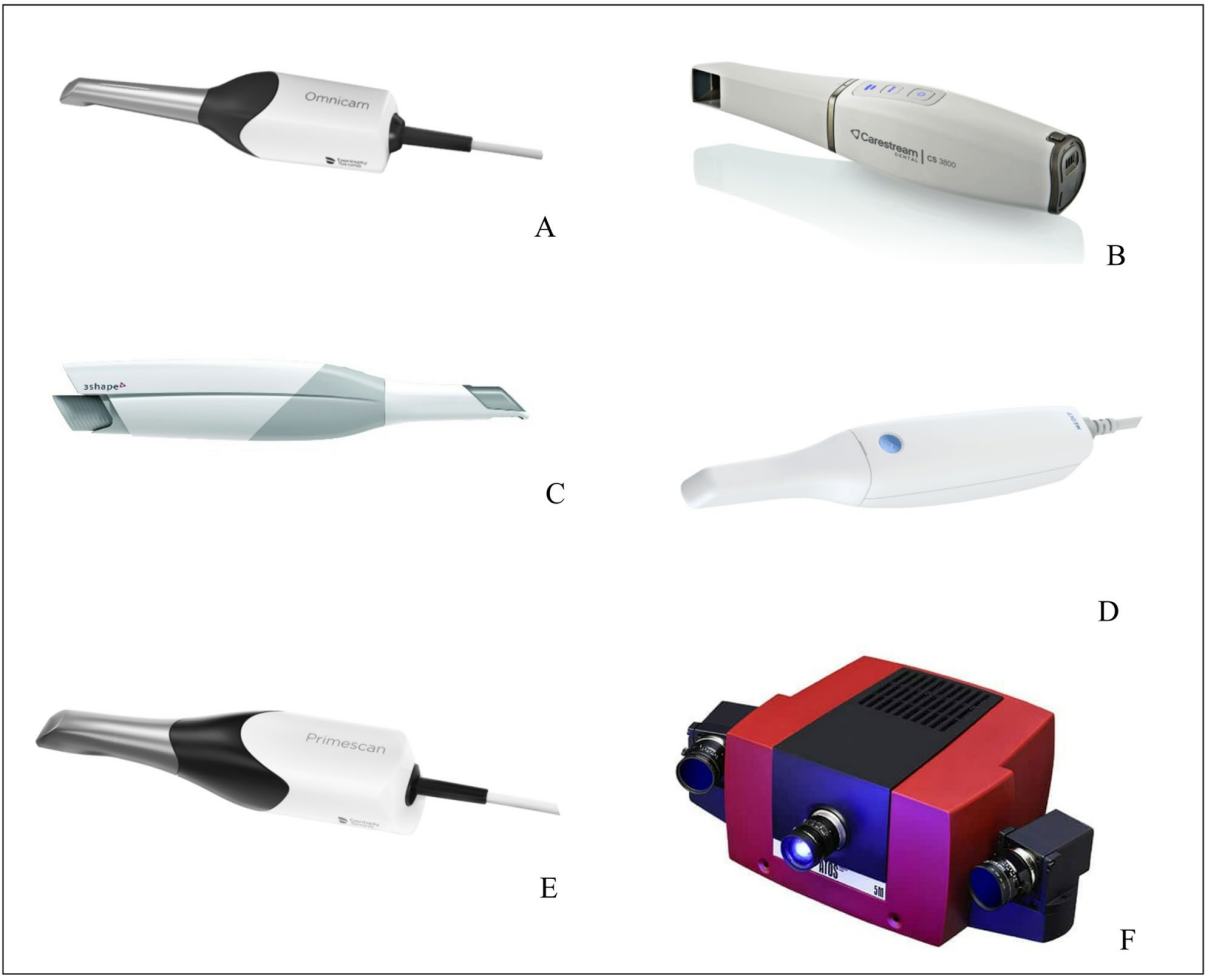
The scanners used in the study. **A**: Cerec Omnicam AF, **B**: CS3800, **C**: 3 Shape Trios 3 A, **D**: Medit i500, **E**: Primescan, **F**: GOM ATOS Compact Scan 5 M

For the purpose of testing their trueness and precision in capturing digital impressions in the field of oral implantology, 5 IOSs, besides the reference industrial scanner, were included in this comparative in vitro study (Fig. 1). The tested IOSs were selected to represent commonly used systems employing different optical acquisition principles. Scanner system, manufacturer, software version and scanning technology for each scanner are listed in Table 1.

**Table 1:** Scanner system, manufacturer, software version, and scanning technology for each scanner used in the study

System	Manufacturer	Software	Technology
Omnicam^®^ AF	Sirona, Bensheim, Germany	Cerec SW5.2.2	Optical triangulation & confocal microscopy.
CS3800	Carestream, Rochester, NY, USA	CS WinOMS 9.1	Active Triangulation
3 Shape Trios 3 A	3-Shape, Copenhagen, Denmark	22.1.10.1	Confocal laser scanning
Medit i500	Medit, Seoul, South Korea	Medit Link 3.0.6	Dual camera optical Triangulation
Primescan AC	Sirona, Bensheim, Germany	CEREC SW 5.2	Confocal microscopy
GOM ATOS Compact Scan 5 M	Carl Zeiss GOM Metrology GmbH, Braunschweig, Germany	GOM Software 2021 Hotfix 5	Blue LED structured light

The printed models with the scan bodies in place were captured using an ATOS industrial desktop scanner, a high-resolution scanner with an accuracy of 2 μm [23]. Three consecutive scans were recorded for each model and imported into 3D inspection and mesh-processing software (GOM Inspect, GOM GmbH), where the three identical scans were averaged for one scan and exported as an STL file to be assigned later as the reference scan.

Five distinct groups were created based on the digitization device, one group for each scanner, and each group had 10 test scans. For all IOSs, the continuous zig-zag scanning technique was implemented [24]. The scan was started from the most distal anatomical landmark (retromolar pad or last molar) in the right quadrant and ended at the most distal anatomical landmark in the left quadrant. All scans were captured under the same lightning conditions and in a room with a temperature of 25 °C. During scanning, teeth, scan bodies, and edentulous ridges were considered as areas of interest.

All intraoral scans were performed by two calibrated operators (M.E. and M.A.) with over 10 years of experience in digital dentistry. Both operators underwent standardized training and performed practice scans on similar models prior to data collection to ensure familiarity with the scanners before a standardized and predefined scanning protocol was followed. All the IOSs tested were calibrated before the first scan following the manufacturer’s recommendations. Additionally, to ensure optimum accuracy, all the scans were recorded without rescanning or stitching procedures, and scans exhibiting interruptions or faults were excluded.

### Three-dimensional 3D data acquisition and evaluation

**Fig. 2:**
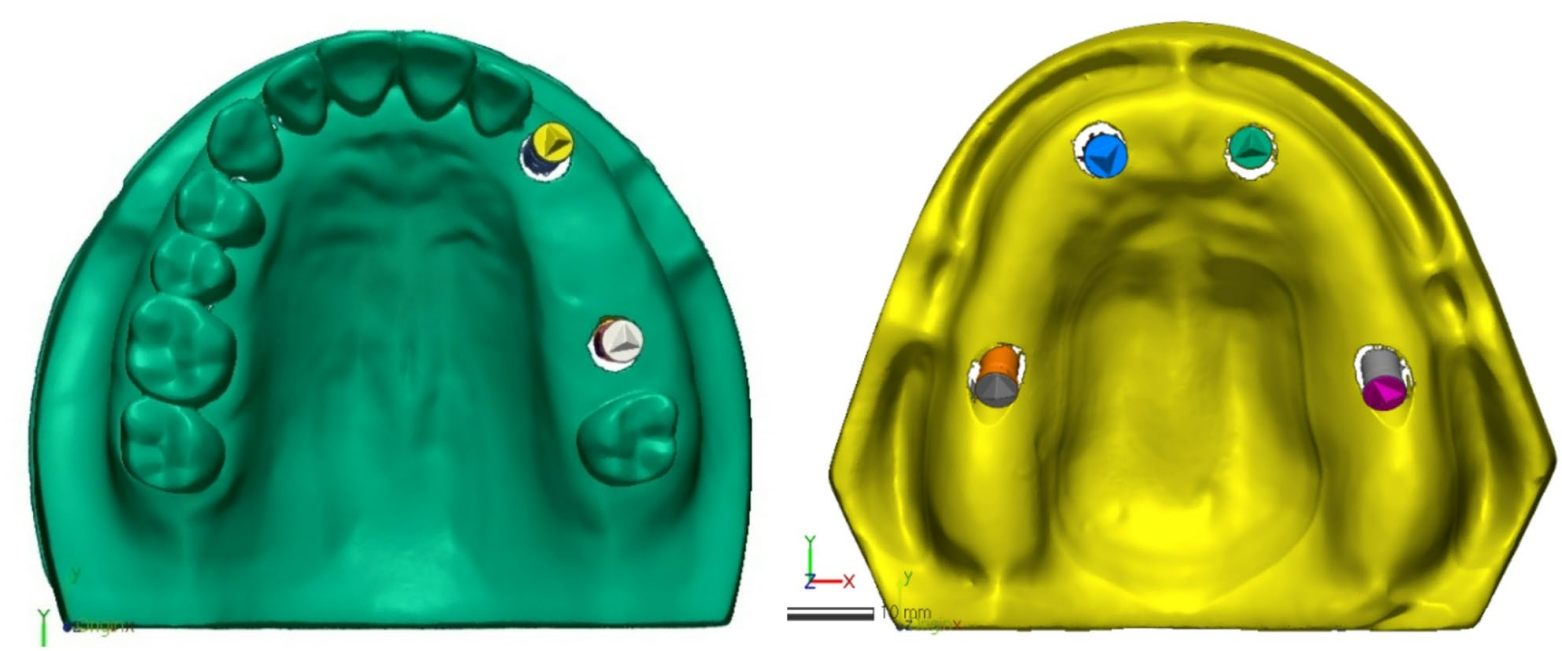
Reference scans segmented to assign scan bodies as different regions from the model

After the completion of the scanning process, all 3D measurements and alignment procedures were conducted using an image analysis software program (Geomagic Control X 2020; 3D Systems) by one calibrated investigator (M.E.) who was blinded to scanner group codes to minimize bias. The investigator received training on the software and established reproducibility through repeated measurements on pilot scans prior to data collection. The reference scan was then imported and assigned as reference data. For 3D comparison, the top surfaces of the scan bodies were used, whereas the remaining regions of the model were merged for alignment purposes (Fig. 2).

**Fig. 3:**
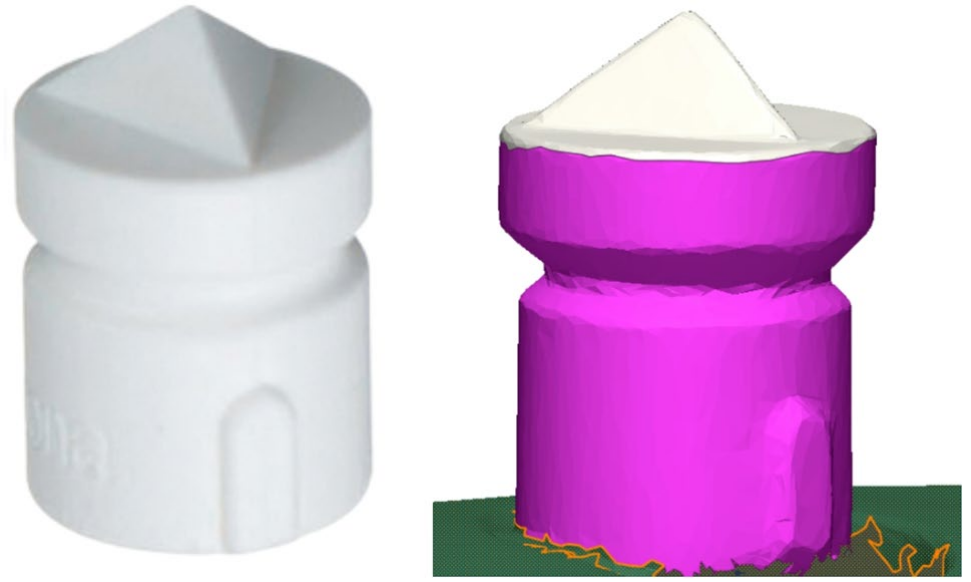
The scan body used in the study with the top surface separated in the STLs for the 3D comparisons

By implementing the functions of “Merge” and “Split” tools in the “Region” tab, the reference scan was segmented to separate the top of scan bodies into separate regions. Segmentation was performed semi-automatically, following a standardized protocol for all scans. Specifically, the top surface of each scan body was first automatically selected by the software’s region detection algorithm, which identifies surface curvature discontinuities, and was then manually verified and adjusted as needed to ensure consistent boundary definition (Fig. 3).

Assessing trueness typically involves comparing the intraoral scans to the reference dataset obtained using a high-resolution scanner with a known accuracy of < 5 μm [8, 23]. Accordingly, each test scan in this study was imported individually into the analysis software, where it was aligned with the scan acquired using the industrial scanner. This process was repeated for each group sequentially. In contrast, evaluating precision simply involves comparing multiple scans captured using the same IOS to determine consistency [8]. It was evaluated by superimposing the STL files obtained from each scanner within the same group. For each scanner, the 10 test scans were labeled from “1” to “10” and systematically compared in pairs, beginning with scan 1 aligned with scans 2 through 10, then scan 2 with scans 3 through 10, and so forth, ensuring that all possible pairwise combinations were analyzed. The alignment was performed using the software’s “initial alignment” followed by the “best-fit alignment” functions. Notably, the scan bodies were excluded during the alignment process and were subsequently analyzed for deviation using the 3D comparison function.

Following alignment, the 3D comparison tool was used to quantify the deviation at each point of interest on the top surfaces of the isolated scan body regions. Color-coded deviation maps were generated, where blue indicated an inward (negative) deviation and red indicated an outward (positive) deviation of the test scan, thereby visually representing the extent and distribution of the discrepancies across the scan bodies [3]. Deviation values were expressed as root mean square (RMS), which was automatically computed by the software. This calculation involved squaring the distance between corresponding data points, averaging these values over the total number of points, and taking the square root [25]. Lower RMS values corresponded to smaller deviations, indicating greater similarity between aligned scans and, consequently, higher trueness or precision. Conversely, higher RMS values reflected larger deviations, reduced similarity, and lower trueness or precision.

### Statistical analysis

Continuous data were presented as mean and standard deviation (SD) values. ANOVA models were built to study the effect of different variables on the tested outcomes. Models’ residuals were explored for normality by visual inspection and using the Shapiro-Wilk test. Due to the violation of normality assumptions (Shapiro-Wilk test, *p* < 0.05) and heterogeneity of variance (Levene’s test, *p* < 0.05) in the RMS trueness and precision datasets, a Box-Cox transformation was applied to both outcome variables. The estimated lambda parameters were 0.38 for trueness and 0.08 for precision in the three-way models, -0.28 for trueness and -0.01 for precision in the two-way model. Following transformation, both normality (Shapiro-Wilk test, *p* > 0.05) and homogeneity of variance (Levene’s test, *p* > 0.05) assumptions were satisfied.

All inferential statistical analyses were conducted on Box–Cox–transformed data to satisfy ANOVA assumptions. Descriptive statistics (means and standard deviations) are reported on the original measurement scale to facilitate interpretation of RMS values. Post hoc comparisons of estimated marginal means were performed using the model-specific error term, with Holm adjustment applied to control for multiple testing.

A three-way ANOVA was selected to simultaneously evaluate the main effects of scanner type, restoration type, and implant position, as well as their interactions, which is essential for understanding how these factors jointly influence accuracy in clinical implant scanning scenarios. The significance level was set at *p* < 0.05 within all tests. Statistical analysis was performed with R statistical analysis software version 4.5.1 for Windows (R Core Team, 2025) [26].

## Results

Statistically significant three-way and two-way interaction effects were identified among scanner type, restoration configuration, and implant position. Therefore, scanner-related differences in trueness and precision are presented and interpreted within specific restoration types and implant positions, rather than as generalized scanner-specific effects.

### Trueness

The results of the three-way ANOVA indicated statistically significant main effects of restoration type, scanner type, and implant position on the trueness of digital impressions (*p* < 0.001). The largest effect size was observed for the scanner type (PES = 0.9), followed by implant position (PES = 0.8), and restoration type (PES = 0.1). All interaction effects between the three variables were significant in three- and two-way ANOVA (*p* < 0.001).

**Fig. 4:**
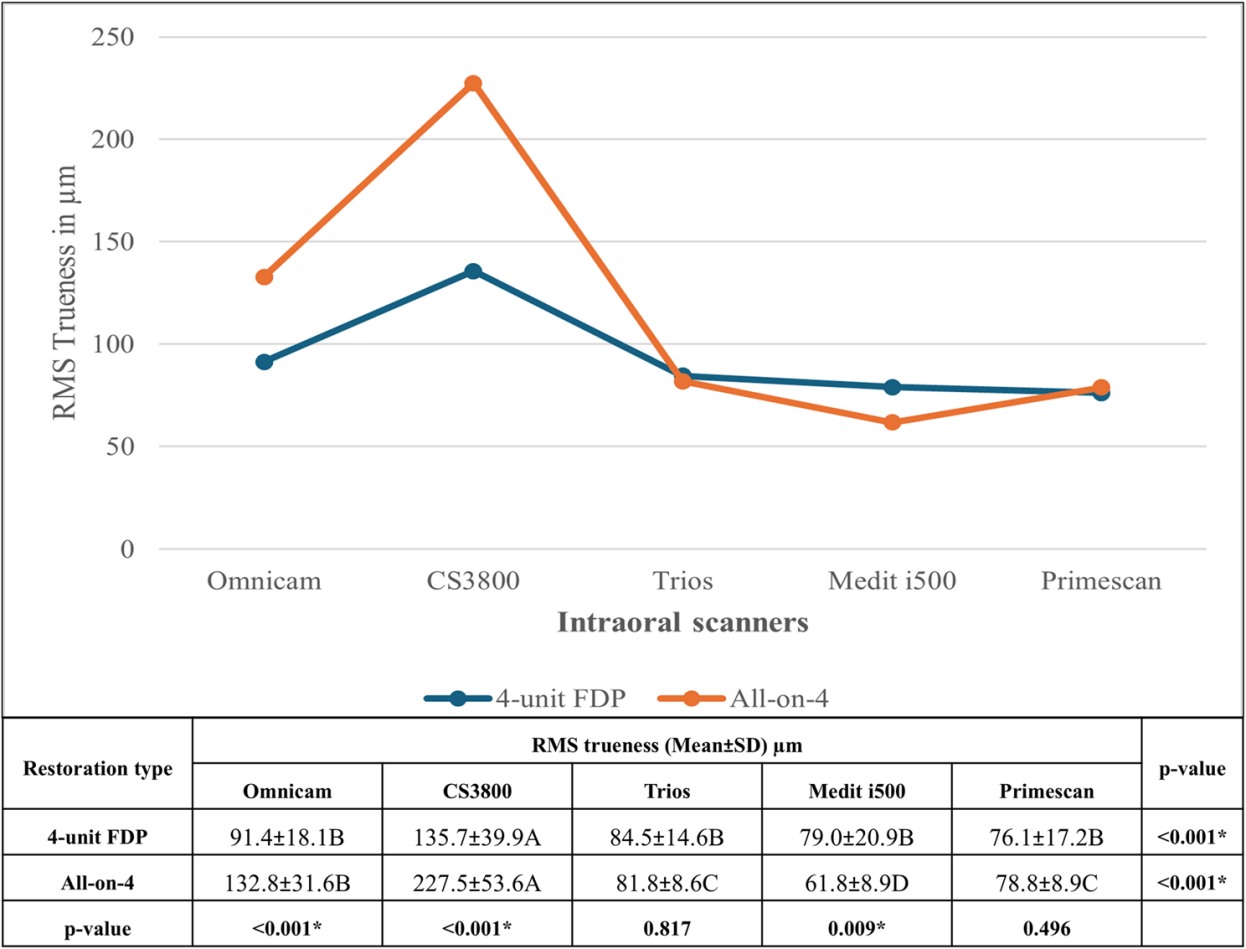
Comparison of RMS trueness of intraoral scanners for 4-Unit FDP and All-on-4 Restorations (Mean ± SD, µm). Values with *different letters* within the *same horizontal row* are significantly different; * significant (*p* < 0.05)

Post-hoc comparisons showed that for all implants combined in the 4-unit FDP restoration type, there was a non-significant difference between the scanners in RMS trueness values, except for CS3800, which showed significantly higher RMS values than the other scanners. While for All-on-4, Medit i500 showed the least RMS trueness values and CS3800 the highest. Moreover, Omnicam and CS3800 -in 4-unit FDP scans- showed significantly lower RMS values than All-on-4. The comparison of RMS Trueness of Intraoral Scanners for 4-Unit FDP and All-on-4 Restorations are shown in Fig. 4.

**Fig. 5:**
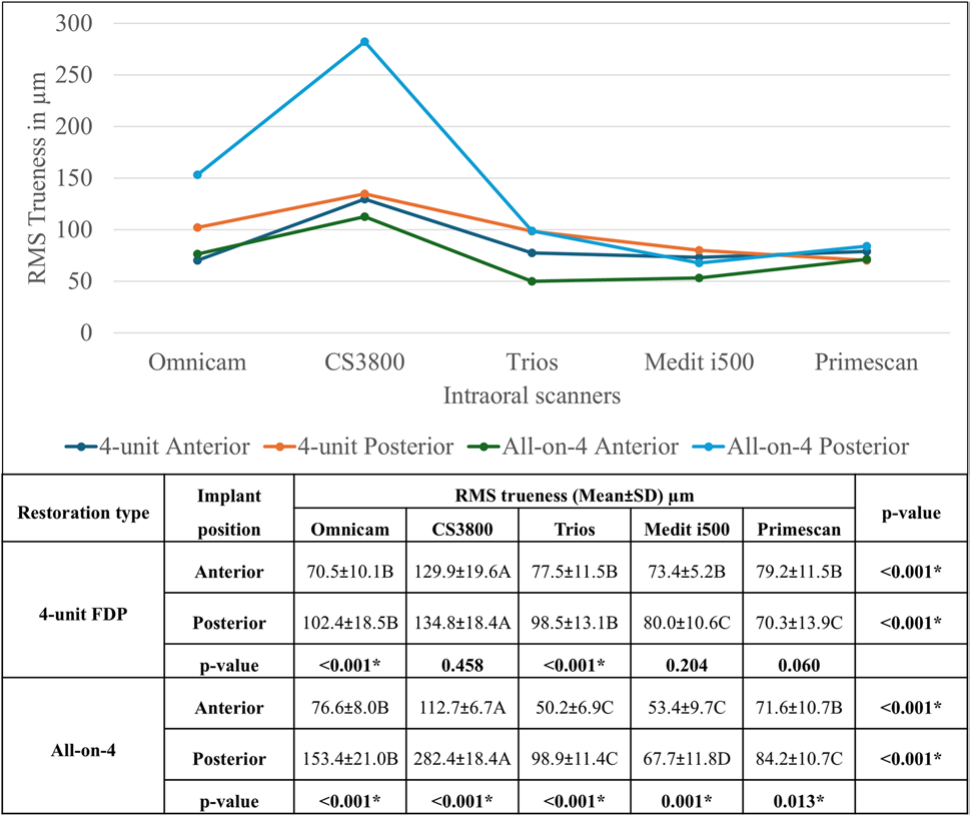
Comparison of RMS trueness (Mean ± SD, µm) of intraoral scanners by restoration type and implant position. (Values with *different letters* within the *same horizontal row* are significantly different; * significant (*p* < 0.05))

In the 4-unit FDP group, RMS values for trueness were significantly higher in both anterior and posterior implant positions for CS3800 compared to other scanners. In the All-on-4 group, similar findings were noted where Trios 3 and Medit i500 demonstrated significantly lower RMS values in anterior implant positions and Medit i500 in posterior implant positions. Whereas CS3800 showed the highest RMS values in both the anterior and posterior regions. In the 4-unit FDP group, Omnicam and Trios showed significantly higher posterior implant RMS values compared to anterior implant. Within the All-on-4 group, RMS values for posterior implants were significantly higher than those for anterior implants, regardless of the scanner used (Fig. 5).

**Fig. 6:**
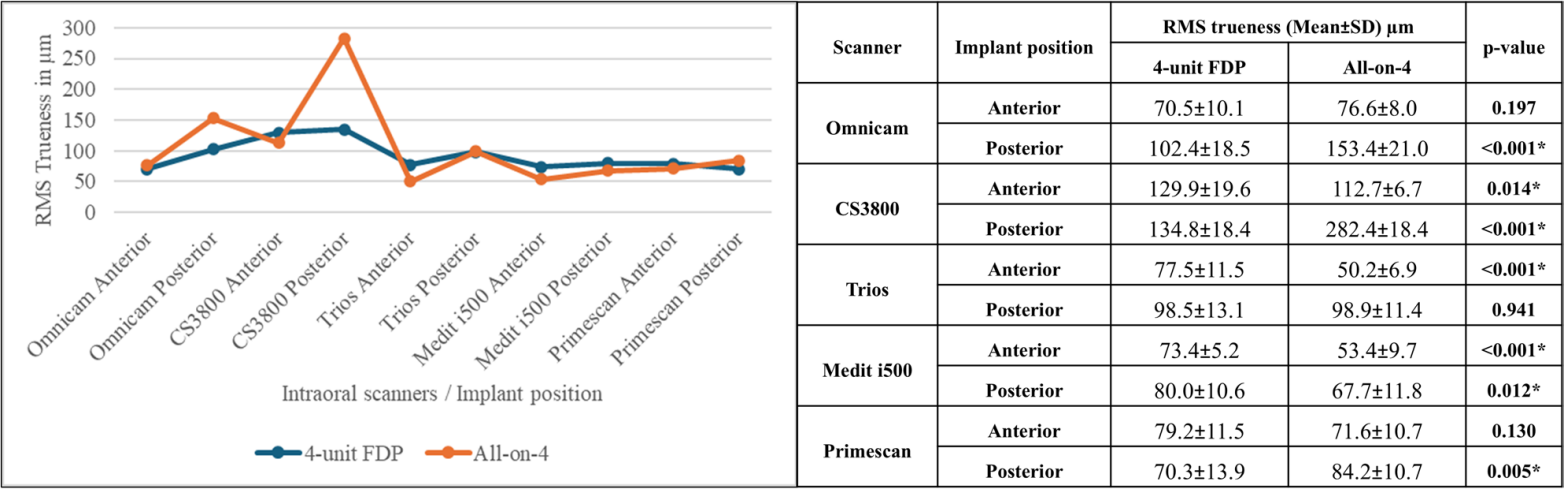
Comparison of RMS trueness of intraoral scanners for 4-Unit FDP vs. All-on-4 by implant position (Mean ± SD, µm). *: Significant (*p* < 0.05)

RMS trueness values of anterior implants positions in 4-unit FDPs were statistically significantly higher than anterior implants in All-on-4 for all scanners except for Omnicam and Primescan, as it was non-significant. Moreover, the RMS trueness of posterior implants position was statistically significantly lower in 4-unit FDP than in posterior implants in All-on-4 for all scanners except Trios 3, where the difference was insignificant, and Medit i500, which showed higher RMS values for posterior implants in 4-unit FDP (Fig. 6).

### Precision

Three-way ANOVA results demonstrated statistically significant effects of restoration type, scanner type, and implant position on precision (*p* < 0.001), with implant position showing the most substantial effect (PES = 0.9), followed by scanner type (PES = 0.8). All interaction effects were statistically significant. Two-way ANOVA results for overall precision also confirmed significant effects for scanner type and its interaction with restoration type (*p* < 0.001), while the effect of restoration type alone was not significant (*p* = 0.9).

**Fig. 7:**
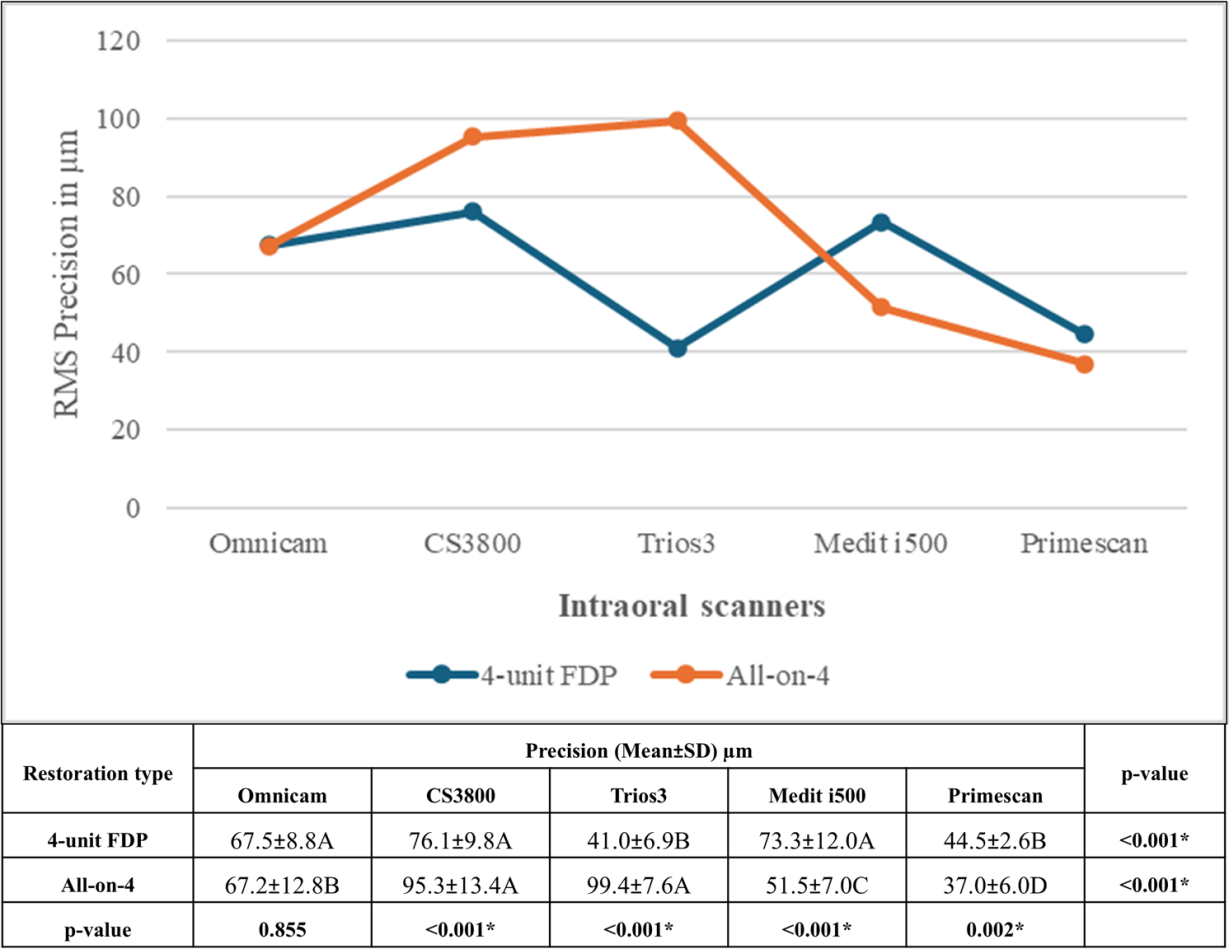
Comparison of RMS precision of intraoral scanners for 4-Unit FDP and All-on-4 restorations (Mean ± SD, µm). Values with *different letters* within the *same horizontal row* are significantly different; * significant (*p* < 0.05)

Regarding interaction between scanner type and restoration type, post-hoc comparisons revealed that precision values confirmed statistically significant differences among scanners for both the 4-unit FDP and All-on-4 restorations (*p* < 0.0). Specifically, in the 4-unit FDP group, Trios 3 and Primescan exhibited significantly lower RMS values compared to other scanners. Conversely, in the All-on-4 group, Primescan exhibited significantly the lowest RMS values, while Trios3 and CS3800 recorded the highest (Fig. 7).

**Fig. 8:**
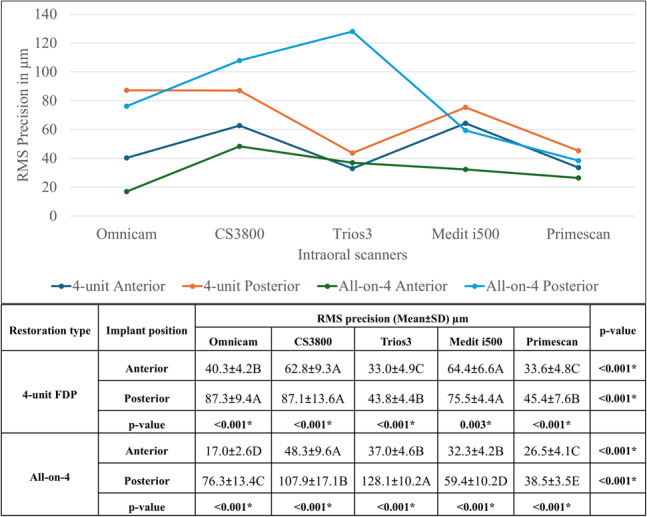
Comparison of RMS precision of intraoral scanners by restoration and implant position (Mean ± SD, µm). Values with *different letters* within the *same horizontal row* are significantly different; * significant (*p* < 0.05)

In the 4-unit FDP group, Trios3 and Primescan achieved the lowest RMS precision values in both implant positions. In All-on-4, Omnicam had the least RMS values in anterior implants, while Primescan had the least RMS in posterior implants. All scanners showed significantly less precision in posterior implants in both restoration types (Fig. 8).

**Fig. 9:**
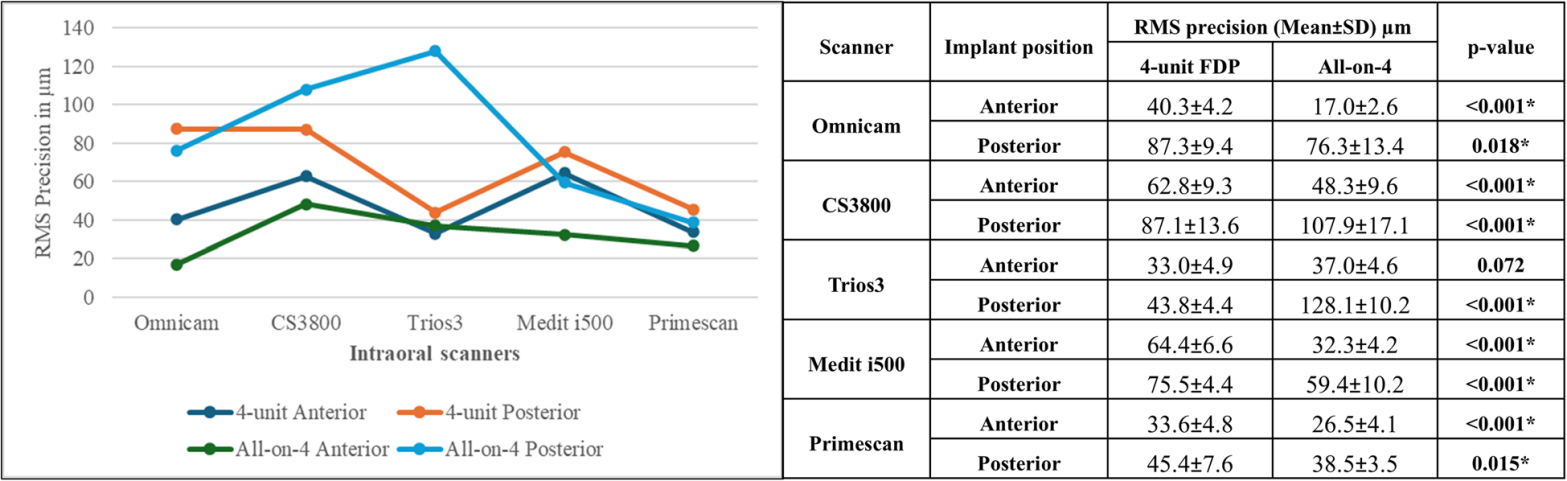
Comparison of RMS precision of intraoral scanners for 4-Unit FDP vs. All-on-4 by implant position (Mean ± SD, µm). *: Significant (*p* < 0.05)

RMS precision values of anterior implants in the 4-unit FDP were statistically significantly higher than in All-on-4 for all scanners except Trios3, which showed a non-significant difference (*p* = 0.1). Furthermore, the RMS precision values of posterior implants were significantly higher in the 4-unit FDP than in All-on-4 for all scanners except for CS3800 and Trios3 (Fig. 9).

## Discussion

Previous studies indicate that IOS accuracy varies considerably with scanning span and anatomical location, with full-arch and edentulous situations presenting greater challenges than dentulous conditions [14, 18, 24, 27]. This study aimed to comprehensively evaluate the trueness and precision of five intraoral scanners across two clinically relevant implant restoration situations: 4-unit FDPs and full-arch All-on-4 restorations, further dissected by implant position (anterior vs. posterior). The tested IOSs were deliberately selected to represent different optical acquisition principles, manufacturers, and cost categories. The study findings underscore the complexity of digital impression accuracy, which varies by scanner technology, edentulous span length, and anatomical position. As all interaction effects between the three variables were significant in three-way ANOVA (*p* < 0.001), the null hypothesis was rejected, and the interpretation that scanner technology is considered a contributing factor rather than a sole determinant of trueness and precision was supported.

For the 4-unit FDP configuration, trueness values were comparable among scanners, with CS3800 demonstrating lower trueness relative to the remaining systems. In contrast, for the All-on-4 configuration, scanner-dependent differences became more pronounced, with Medit i500 exhibiting higher and CS3800 lower trueness. These findings suggest that scanner-related differences may become more evident as restoration span increases.

Mangano et al. [8] reported lower trueness values for CS3500 and Trios2 scanners when evaluating partially and fully edentulous implant models. While these results differ from those observed in the present study, direct comparison is limited by important methodological differences in scanner generations, implant configurations, and deviation analysis protocols. Notably, Mangano et al. [8] assessed deviations across the entire arch, whereas the present study focused on scan body-based analysis, which may differentially influence calculated trueness outcomes.

Several previous investigations have reported scanner-dependent differences in trueness for both short-span and full-arch implant restorations [18, 24]. Through these studies, Carestream and Trios systems frequently demonstrated reduced deviations, and consequently higher trueness, in partially edentulous configurations. Whereas increased deviations, indicating reduced trueness, were commonly observed in full-arch scans. Despite variations in the scanners evaluated and the magnitude of deviations reported, the overall reduction in trueness with increasing scan span is consistent with the patterns observed in the present study.

Consistent with previous studies [18, 24], trueness in the present study was generally higher for 4-unit FDPs than for All-on-4 restorations across all tested scanners. This finding may be attributed to the reduced availability of anatomical landmarks and the increased stitching complexity in edentulous arches, which have been widely recognized as major contributors to cumulative error during digital scanning [28].

The comparatively lower trueness deviations observed with the Medit i500 in the All-on-4 configuration may be related to scanner-specific data visualization and real-time feedback features that assist operators in identifying incomplete or unreliable scan regions during acquisition. In particular, the “model display mode,” which converts mesh areas from red to green during scanning, allows the operator to detect and avoid distortion or double imaging. Additionally, the “reliability map” overlays color-coded information onto the model to indicate data quality, with green representing high reliability and red indicating poor reliability. Although these software tools may enhance data consistency during scanning, their isolated effect on trueness could not be determined within the scope of the present study.

In the present study, within the 4-unit FDP configuration, posterior implants demonstrated lower trueness than anterior implants when scanned with Omnicam and Trios systems. Similar positional effects have been reported in previous in vitro investigations, in which increased deviations were observed at implant sites captured later in the scanning sequence, likely due to cumulative distortion arising from stitching procedures during data acquisition. Additionally, the presence of a non-anatomical edentulous span preceding the posterior implant may have further contributed to the observed posterior discrepancies [29].

In our study, posterior tilted implants (45°) in the All-on-4 configuration exhibited greater deviations than anterior parallel implants across all scanners. This finding is consistent with studies suggesting that implant angulation may negatively influence scan accuracy in complete-arch digital impressions [30]. However, other investigations have reported minimal or no influence of angulation on digital impression accuracy [31, 32]. These inconsistencies likely reflect differences in implant distribution, reference geometry, and analytical methods rather than true disagreement among findings.

In the present study, the trueness of anterior implant positions in the 4-unit FDP configuration was lower than that of anterior implants in the All-on-4 configuration for most scanners. Van der Meer et al. [33] and Ahlholm et al. [2] attributed reduced trueness in more distal regions to the accumulation of registration errors during scan progression and image stitching. Accordingly, because the anterior implant in the partially edentulous maxilla model was positioned more posteriorly than the anterior implants in the All-on-4 model, it may have been more susceptible to cumulative stitching errors, resulting in greater positional deviation.

Moreover, posterior implant trueness was higher in the 4-unit FDP configuration than in the All-on-4 configuration for all scanners, except Trios and Medit i500. This difference may be attributed to variations in posterior implant angulation between the two models, as discussed earlier. Regarding precision, Trios 3 and Primescan demonstrated lower deviations in repeated scans and, consequently, higher precision in the 4-unit FDP configuration, consistent with previous studies evaluating short-span implant restorations [34]. In contrast, certain scanners, such as Trios 3 and CS3800, exhibited lower precision in the All-on-4 configuration, reflecting greater susceptibility to cumulative error in extended scanning scenarios. The maintained high precision observed with Primescan across larger spans may be attributed to their advanced capturing technologies and software enhancements designed to minimize stitching or registration errors, as previously reported by Mandelli et al. [35].

Imburgia et al. [24] reported no statistically significant scanner-dependent differences in precision between partially and fully edentulous models. Differences between their findings and the present results may be attributed to variations in scanner versions, reference alignment strategies, and precision calculation methods, underscoring the challenge of directly comparing precision outcomes across studies. Moreover, Miyoshi et al. [36] examined the influence of scanning range on the precision of digital impressions using an edentulous maxillary model with six implants scanned by four IOSs. Precision was evaluated through 3D superimposition across nine predefined scanning ranges corresponding to different implant spans. Their results demonstrated a consistent decrease in precision as the scanned area increased, indicating that scan extent is a critical factor influencing accuracy. These findings support the observation that shorter scanning spans are associated with reduced cumulative error [36].

Across both restoration configurations in the present study, posterior implants consistently demonstrated lower precision compared to anterior implants. This finding indicates a positional effect on scan repeatability under controlled in vitro conditions. However, the observed differences reflect experimental accuracy outcomes and do not establish clinical thresholds, prosthesis fit implications, or in vivo scanning recommendations. Moreover, the precision of anterior implants in the 4-unit FDP was lower than that observed in All-on-4 across all scanners. This difference can likely be attributed to cumulative errors that increase as the scanning process progresses along the arch, resulting in reduced accuracy for implants scanned later in the sequence.

A previous comparative study evaluating 14 IOSs within an All-on-4 workflow reported scanner-dependent differences in precision outcomes [37]. While variability among devices was identified, direct comparison with the present findings is limited by differences in scanner selection, analytical metrics, and experimental conditions. Moreover, the absence of a consistent precision hierarchy across studies suggests that observed differences are context-dependent rather than indicative of universally superior or inferior scanner performance.

Comparisons with previous studies are challenging, not only due to differences in research methodologies but also because of variations in measurement techniques. Some studies evaluated scan accuracy by comparing linear and angular deviations between IOS scans and reference scans, often expressed using the mean absolute deviation (MAD). In contrast, other studies superimposed the 3D surface data obtained from the IOS and reference scanner and calculated discrepancies using root mean square (RMS) values [38]. RMS has gained increasing popularity as a metric for evaluating accuracy because it provides comprehensive information on 3D deviations. However, RMS values are generally higher than MAD values, which further complicates comparisons. For example, in a previous study [39], the average MAD for trueness was 101.27 μm, in case of the CEREC Omnicam scanner, whereas the corresponding RMS value -in the same study- nearly doubled to 197.9 μm.

Interestingly, previous research [40] has indicated that the accuracy of digital impressions is not necessarily determined by the resolution of data acquisition. In implantology, mesh density-reflected by the number of triangles-plays a less critical role than overall positional accuracy, as the primary objective is to capture the precise location of the implant. In contrast, for natural teeth, higher acquisition resolution is advantageous because it enhances the visibility of the prosthetic preparation margin. This suggests that, although the stitching process during scanning can influence accuracy, the resulting deviations across the entire arch generally remain within acceptable limits for producing high-quality scans suitable for prosthetic workflows. Currently, no universally accepted clinical threshold for IOS accuracy exists, and tolerance to deviation may vary according to prosthetic design and clinical scenario. Previous studies have proposed a wide range of reference values for IOS accuracy [11, 41, 42]; however, no consensus has been reached. Accordingly, the clinical significance of the observed differences depends on the specific prosthetic workflow, restoration design, and individual case requirements, rather than representing absolute suitability or unsuitability [39].

This study has several limitations. First, as an in vitro investigation, it cannot fully replicate the complex conditions of the oral environment, including limited light, humidity, soft tissue movement, and the presence of saliva. Additionally, scanning plaster models is inherently easier than intraoral scanning due to greater accessibility and optical differences. Second, there is a potential violation of the independence assumption, as multiple scans and implant positions were obtained from the same two physical models. Although each scan was performed independently, shared model characteristics may have introduced some correlation among observations. Third, while the latest software versions were used, future updates could improve scanning accuracy, and therefore, the results are specific to the scanner models and software versions tested. Finally, clinical studies are needed to evaluate how differences in digital impressions impact prosthesis fit, function, and patient satisfaction, and future studies employing multiple models or mixed-effects analyses to account for within-model clustering would further strengthen the statistical approach.

## Conclusion

Within the limitations of this study, the following conclusions can be drawn.

1. The accuracy of digital impressions was influenced by scanner type, restoration configuration, and implant position.
2. In 4-unit restorations, CS3800 demonstrated lower trueness compared to other scanners, while Trios3 and Primescan showed higher precision in anterior implant positions.
3. For All-on-4 restorations, both the Trios3 and Medit i500 demonstrated higher trueness for anterior implants, while Medit i500 showed higher trueness for posterior implants. Conversely, CS3800 showed the lowest trueness.
4. Scanning of posterior implant positions generally exhibited less precision than anterior positions for all scanners and across both restoration configurations.
5. These study findings are based on in vitro conditions and should be interpreted within the context of the experimental model used.

## Supplementary Information

Supplementary Material 1.

## Data Availability

The data that support the findings of this study are available from the corresponding author upon reasonable request.

## Abbreviations

IOS: Intraoral scanner
3D: Three dimensional
CAD/CAM: Computer-aided design/ Computer-aided manufacturing
µm: Micrometer
FDP: Fixed Denture Prosthesis
STL: Standard Tessellation Language
°C: Degrees Celsius
Ncm: Newton centimeters
RMS: Root mean square
PEM: Partially edentulous model
FEM: Fully edentulous model
MAD: Mean absolute deviation
