# Supplementary figures and images for: Accuracy of digital impressions for implant fixed dental prostheses in partial and complete maxillary edentulous arches: effect of intraoral scanners and implant position
Source: BMC Oral Health. 2026 Mar 11;26:610. doi: 10.1186/s12903-026-07887-6 (PMC13063661; doi:10.1186/s12903-026-07887-6)

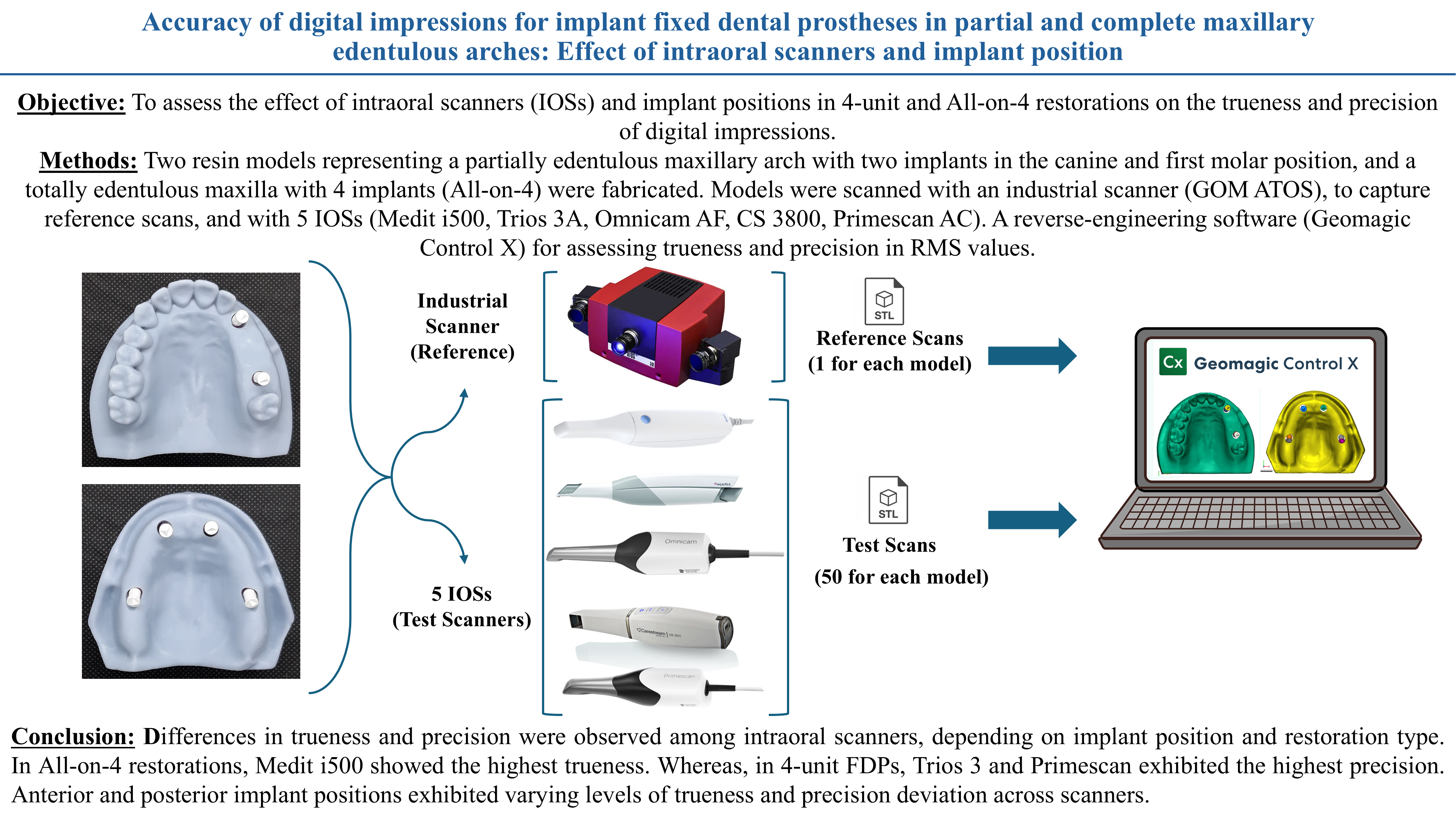

Supplement: Supplementary file 1 — Supplementary Material 1. [file 12903_2026_7887_MOESM1_ESM.tif]
